# Illness beliefs and the sociocultural context of diabetes self-management in British South Asians: a mixed methods study

**DOI:** 10.1186/s12875-015-0269-y

**Published:** 2015-05-10

**Authors:** Neesha R Patel, Carolyn Chew-Graham, Christine Bundy, Anne Kennedy, Christian Blickem, David Reeves

**Affiliations:** 1 Centre for Endocrinology and Diabetes and Manchester Centre for Health Psychology, Institute of Human Development, The University of Manchester, Room S42, Second Floor, Zochonis Building, Brunswick Street, Manchester, M13 9PT UK; 2 Primary Care and Health Sciences, and NIHR Collaboration for Leadership in Applied Health Research and Care (CLAHRC) West Midlands, Research Institute, Keele University, Keele, Staffordshire ST5 5BG UK; 3 Institute for Inflammation and Repair, The University of Manchester, 1.530 Stopford Building, Oxford Road, Manchester, M13 9PL UK; 4 NIHR CLAHRC Wessex, Faculty of Health Sciences, Building 67, University of Southampton, Highfield Campus, Southampton, SO17 1BJ UK; 5 NIHR CLAHRC Greater Manchester, The University of Manchester, Centre for Primary Care, 5th Floor Williamson Building, Oxford Road, Manchester, M13 9PL UK; 6 Centre for Primary Care, The University of Manchester, 5th Floor Williamson Building, Oxford Road, Manchester, M13 9PL UK

**Keywords:** British South Asians, Illness beliefs, Social Networks, Fatalism, Sociocultural, Diabetes, Self-management

## Abstract

**Background:**

British South Asians have a higher incidence of diabetes and poorer health outcomes compared to the general UK population. Beliefs about diabetes are known to play an important role in self-management, yet little is known about the sociocultural context in shaping beliefs. This study aimed to explore the influence of sociocultural context on illness beliefs and diabetes self-management in British South Asians.

**Methods:**

A mixed methods approach was used. 67 participants recruited using random and purposive sampling, completed a questionnaire measuring illness beliefs, fatalism, health outcomes and demographics; 37 participants completed a social network survey interview and semi-structured interviews. Results were analysed using SPSS and thematic analysis.

**Results:**

Quantitative data found certain social network characteristics (emotional and illness work) were related to perceived concern, emotional distress and health outcomes (p < 0.05). After multivariate analysis, emotional work remained a significant predictor of perceived concern and emotional distress related to diabetes (p < 0.05). Analysis of the qualitative data suggest that fatalistic attitudes and beliefs influences self-management practices and alternative food ‘therapies’ are used which are often recommended by social networks.

**Conclusions:**

Diabetes-related illness beliefs and self-management appear to be shaped by the sociocultural context. Better understanding of the contextual determinants of behaviour could facilitate the development of culturally appropriate interventions to modify beliefs and support self-management in this population.

**Electronic supplementary material:**

The online version of this article (doi:10.1186/s12875-015-0269-y) contains supplementary material, which is available to authorized users.

## Background

In the UK, Type 2 diabetes (T2D) and its associated complications are disproportionately high among British South Asians compared to the general UK population [1,2]. T2D is caused by the body’s ineffective use of insulin and has been linked to structural factors including; age, ethnicity and genetic pre-disposition and lifestyle factors including obesity and physical inactivity [1]. There is no one explanation for the increased prevalence of T2D in South Asians; factors such as diet, migration from the Indian subcontinent and physical inactivity are implicated [1].

The management of diabetes poses one of the most challenging regimens of any long-term condition due to the number and complexity of tasks involved in maintaining optimum blood sugar levels [3]. Whilst a number of barriers have been reported to impede optimal self-management in South Asians [4]^,^ fatalistic beliefs, cultural and social norms and their influence on diabetes-related beliefs [5-8] have received most interest in the literature.

Understanding beliefs about diabetes are important to know how people make sense of and manage their illness [9]. A theoretical model that is useful in describing and understanding patterns of beliefs and predicting behaviours related to self-management of diabetes is the Common Sense Self-Regulatory Model (CS-SRM) [10]. According to the CS-SRM, people develop implicit beliefs (cognitions) and emotions about their illness, which consist of five key dimensions: i) identity: perception of the label and symptoms of the illness, ii) timeline: duration of the illness, iii) consequences, and iv) cause: perceptions of the cause of the illness v) cure/control: perceptions of cure/controllability. These cognitions help to guide the management of health threats, cope with symptoms and diagnosis of an illness and health information [11,12].

Research using the CS-SRM has found that beliefs about diabetes play an important role in instigating illness management [13-15], as well as informing interventions to either change or challenge beliefs [16,17]. However, most of the research using the CS-SRM has been conducted with Caucasians and Europeans [18,19]. Qualitative studies have provided insights into the causal beliefs about diabetes and self-management behaviours in this population [7,20]. For example, controlling the intake of sugar, following information and/or advice provided by the General Practitioner (GP) and fatalistic beliefs (i.e. attributing diabetes control to external forces such as God and to believe that deities are more powerful in controlling health and illness) have been related to self-management of diabetes [7] but these factors require further exploration within the CS-SRM domain.

Successful self-management requires an understanding of diabetes. However, cultural health beliefs and poor understanding of diabetes have been reported to impede self-management practices [21-23] resulting in poor diabetes outcomes [24]. Culturally tailored diabetes education programmes designed to improve diabetes self-management in British South Asians have had limited success in improving diabetes outcomes [25]. Given the elevated risk of diabetes in this population, a more complete explanation is needed to better understand the underlying illness beliefs about diabetes and self-management in this population. To date, qualitative research has not captured the influence of the emotional and behavioural responses related to diabetes management, and the quantitative measures do not reflect on the sociocultural factors related to diabetes management [26]. Illness management is often a shared task involving patients’ social networks (e.g. family and friends), [27-29] and it is likely that beliefs about diabetes are influenced by this context [30,31]. Whilst strong family ties in the South Asian population have been linked to shaping attitudes, and the provision of information for diabetes, [20,32] there is a lack of understanding of the sociocultural context of disease representation in the CS-SRM.

The aim of this study was to use mixed methods to build on the CS-SRM literature on diabetes and explore the association between illness beliefs (including fatalism), social networks, cultural beliefs and self-management behaviours in British South Asians to answer the following exploratory questions:

1. What social network factors are associated with illness and fatalism beliefs?
2. How do illness and fatalism beliefs relate to self-management behaviours and health outcomes?
3. What social and cultural beliefs influence the experience and practice of diabetes self-management?

Mixed methods studies facilitate a broader and deeper understanding of issues by providing benefits of different methods while compensating for some of their limitations [33]. We used a sequential embedded mixed methods design - a two-phase mixed design [34] whereby the qualitative findings assisted to elaborate and deepen understanding of the quantitative study findings. The two methods of data collection required separate analyses of data sets, with the findings later integrated and compared. This ‘concurrent triangulation’ [35] approach is a useful way of establishing rigour and credibility of the study findings in mixed methods research [33].

## Methods

This study was conducted as part of The National Institute for Health Research (NIHR) Collaboration of Applied Leadership in Health and Care People with Long-Term Conditions programme (CLAHRC) [36]. Ethical approval was granted from the North West Research Ethics committee – Greater Manchester Central, REC reference: 10/H1008/1. All participants gave informed written consent to take part in the study and received £15 in gift vouchers for their time.

### Recruitment

Two stages of sampling were used. First, n = 30 British South Asian patients with diabetes were randomly selected from the disease registers of 22 consenting GP practices in areas of the North-West of England between April 2010 and January 2011. Second, for the nested qualitative study a purposive sampling method (n = 37 from the local community) was used to increase the sample size, representation and statistical power for the quantitative analysis.

### Power calculation

The sample size was powered at 80% power (at alpha = 5%, with a sample of n = 64) to detect a true correlation of 0.35 or higher between any two variables.

### Quantitative study

Once consented (either via telephone or postal form) to participate in the study, the questionnaire was sent to the participants’ home address provided upon consent. Participants were given two options to return their completed questionnaire: 1: post back in the stamped address envelope provided with the questionnaire, or 2: give it to the first author (NP) prior to the start of the qualitative study interview.

### Measures

#### Illness beliefs

The 9-item Brief Illness Perceptions Questionnaire (BIPQ) [37] was used to measure illness beliefs. Five items assess cognitive representations, (i.e. *consequences, timeline, personal control, treatment control and identity);* two items assess emotional representation, i.e. *concerns* and *emotional response;* and one item assesses illness comprehensibility, *understanding*. Responses were given on a zero to ten point Likert scale. The remaining item on the BIPQ is an open-ended causal beliefs question on which individuals rank the three main causes of their illness. The BIPQ has good internal reliability and has been used with a variety of illness groups [37]. A high proportion of participants had comorbid chronic conditions in addition to diabetes. To ensure that responses to the BIPQ (and fatalistic beliefs, below) related primarily to their experience of diabetes, participants were clearly informed through the study information sheet and consent form to respond to the questions in relation to their diabetes.

#### Fatalistic beliefs

Although the Illness Perceptions Questionnaire-Revised [38] is a longer measure of illness beliefs, it only has one item measuring fatalism - ‘chance or bad luck’, and for practical (e.g. time) reasons it was more feasible to use the BIPQ. Two items from a previous study measuring fatalistic beliefs in a multi-ethnic population with diabetes [39] were included, as BIPQ does not measure fatalistic beliefs. Item 1 assesses whether participants believe their diabetes is largely dependent on chance or fate; item 2 assesses whether participants believe there is very little they can do to personally improve their diabetes-related health status. Responses were given on a 5-point Likert scales ranging from 1‘strongly disagree’; 5‘strongly agree’.

#### Diabetes self-care

We used the Summary of Diabetes Self Care Activities scale (SDSCA) [40] to measure the frequency of diabetes-related self-management activities undertaken over the preceding seven days. The measure comprises specific questions relating to general diet, exercise and smoking and has been validated in studies with South Asian people [41,42]. Items on blood-glucose testing and foot care in this measure were excluded as they were not used in the wider CLAHRC sample because it included patients with heart disease as well as diabetes. Each item is measured using a Likert scale ranging from zero days to seven days.

#### Physical health

We used the Short-form 12 (SF12) [43] and applied structural equation modelling (SEM) to obtain oblique (correlated) physical and mental component scores [44]. These scores were obtained using the wider CLAHRC study sample (i.e. Caucasians and South Asians) [29]. The two scores correlated very highly (r = 0.83), indicating strong colinearity. We therefore used the physical component score, (derived from the SEM), as a measure of perceived health status and excluded the mental component score.

*Socio-demographic* data included: gender, age, marital status, employment status and ethnicity.

#### Long-term conditions

Data was collected on length of time (years and months) participants had lived with diabetes and other long-term medical conditions using open-ended questions.

#### Social networks survey interview

Participants who completed the questionnaire were invited to participate in a face-to-face, semi-structured social networks survey interview (SNSI) with the author and lead researcher (NP). Traditional measures of social support and social network analysis has focused on perceived and actual support [45] and less on long-term condition management *per se*. Thus, adopting social network methods from CLAHRC [46,47], participants were asked to map social network members considered to be important for helping to manage their diabetes using a diagram consisting of three concentric circles of decreasing importance [48] (See Vassilev et al. [29] for a detailed description of the network approach adopted). NP provided assistance with this task. We collected characteristics (see Additional file 1) about each social network member and assessed perceived contribution on a Likert scale (from 1 = not at all, to 5 = a lot) of each member to each of the social network characteristics using the questionnaire devised by the CLAHRC study [29]. For full details on network characteristics and variables, see Vassilev et al. [29] and Reeves et al. [49].

#### Nested qualitative study

A separate topic guide was nested in the SNSI to explore in-depth, beliefs about diabetes self-management. The topic guide was informed by the gaps identified in previous research in this field and through discussion with CCG, AK and CB. The interviews took place either at the participant’s home or a preferred location (e.g. community centre), lasting approximately 1–1.5 hours (including time for the below nested qualitative study). Language support was offered to participants prior to the interview. A professional interpreter, independent to the study provided language support for participants whose first language was not English (n = 9). Some participants (n = 6) preferred a member from their family to help with interpretation rather the professional interpreter.

### Quantitative analysis

SPSS v19.0 was used for quantitative analyses. The causal dimension of the BIPQ was analysed using the guidelines provided by Broadbent et al. (2006) [37]. All of the BIPQ and fatalism measures had score distributions that were not normally distributed (Kolmogov-Smirnov test; p < 0.05). The median has been suggested to be a better indicator of central tendency in studies with small sample sizes and less sensitive than the mean to extreme scores [50]. For consistency with previous research using the BIPQ we report item means and standard deviations, but for formal testing of relationships to other factors used non-parametric tests where appropriate. Spearman correlations and Kruskal–Wallis tests were used to examine univariate relationships between variables; but for multivariate analysis parametric forward stepwise multiple regression was used, in the absence of a suitable non-parametric alternative. An α level of 0.05 was used for all statistical analysis. No adjustment was made for multiple testing, as this study was exploratory in nature and did not have a pre-specified set of specific hypotheses. Rates of missing data on the primary measures, BIPQ and fatalism, were very small (maximum of two cases), therefore missing data imputation was not used for these. Rates were greater for some of the demographic measures (up to 25%, for qualifications). In these cases imputation was undertaken using a combination of item mean/mode when the missing data rate was <5%, and regression imputation otherwise. Sensitivity of the results to bivariate outliers was checked by computing Mahalanobis distances for any univariate relationships with a p-value of 0.1 or less. Data-pairs with a Mahalanobis distance significant at p < 0.01 were removed and the analysis repeated [51]. Results for which statistical significance altered after removal of outliers are indicated in the text and tables.

### Qualitative analysis

All interviews were conducted by NP, audio taped, transcribed verbatim, and analysed thematically using principles of grounded theory and constant comparison methods [52]. A full-grounded theory approach was not used as this study had *a priori* assumptions about the study and sample that were influenced by theoretical assumptions and ideas from the literature [52]. Initially open coding was used to analyse the transcripts and, through comparison of these codes, categories and themes were identified. Themes were developed independently by all authors and then agreed through discussion. Field notes and written memos were used to help develop interpretations during analysis. Data collection continued until category saturation was achieved. Atlas.ti6 software was used to store and manage the data.

## Results

### Sample characteristics

Of the n = 67 South Asians who completed the questionnaire, n = 37 completed the SNSI and nested qualitative study due to time and resource constraints but also because mixed methods studies do not always require an equal sample size [34]. The response rate for the number of British South Asian patients invited to take part in study from the GP practices is unknown, as not all GP practices routinely recorded the ethnicity of South Asian patients. Table 1 shows the demographic characteristics of the study sample, which consisted of roughly equal numbers of men and women, with a mean age of 61 and mostly Indian in origin. 84% of participants had at least one other chronic illness in addition to diabetes and this reflects the generally high level of multimorbidity in older diabetic patients [53].

**Table 1:** **Demographic characteristics**

		(n)	%
Total sample		67	100
Gender	Male	36	54
	Female	31	46
Age (mean = 61.0, SD = 12.5)			
Employment	In paid work	18	27
	Retired	28	42
	Other	21	31
Marital status	Married or in civil partnership	54	81
	Other	13	19
Sub ethnic groups	Indian	43	64
	Pakistani	21	31
	Bangladeshi	2	3
	Other (Nepalese)	1	2
Total no of conditions	One or more condition	11	16
	Two or more conditions	56	84
Duration of diabetes	0 to 5 years	14	21
	5 to 10 year	19	28
	10 years +	34	51
Diabetes Type	Type 1	8	12
	Type 2	59	88
Number of cohabitants	No cohabitants	2	5
*(Social networks survey interview only)*	One or more co-habitant	35	95

### Illness beliefs, social network and health outcome measures

Table 2 shows that most participants believed that their diabetes would last forever (mean = 8.91, SD = 2.19) (*timeline*), but many reported low levels of personal control, (mean = 4.22, SD = 2.75), treatment control (mean = 2.89, SD = 2.43), and poor understanding (mean =2.12, SD = 2.21). They were relatively *concerned* (mean = 6.77, SD = 2.92) about the *consequences* of their diabetes (mean = 4.73, SD = 3.07), and experienced some emotional distress (mean =5.73, SD = 3.09). The fatalism scores ranged from 1 indicating least fatalistic beliefs to 5, indicating most fatalistic beliefs. Most tended to believe that their diabetes was largely dependent on chance or fate, (mean = 3.36, SD = 1.48), with a lack of personal control to improve their diabetes, (mean = 2.98, SD = 1.45).

**Table 2:** **Mean and median illness beliefs, fatalism social network and health outcomes scores**

BIPQ	N	MEAN (SD)	MIN	25^th^Percentile	Med	75^th^Percentile	MAX
Consequences	66	4.73 (3.07)	0	2	5	7	10
Timeline	65	8.91 (2.19)	0	9	10	10	10
Personal control	67	4.22 (2.75)	0	2	5	6	10
Treatment control	66	2.89 (2.43)	0	.75	3	5	10
Identity	66	5.35 (2.92)	0	3.75	5	8.00	10
Concern	66	6.77 (3.12)	0	5	8	9.25	10
Understanding	67	2.12 (2.21)	0	0	2	3	9
Emotional distress	67	5.73 (3.09)	0	3	6	8	10
**Fatalism 1** *My illness is largely dependent on chance or fate?*	64	3.36 (1.48)	1	2	4	5	5
**Fatalism 2** *There is very little I can do to personally improve my health status?*	66	2.98 (1.45)	1	1.75	3	4.00	5
**Social Network Factors**							
Mix of network agents	37	3.78 (1.10)	2	3	4	4.5	7
Size of network	37	7.59 (2.73)	3	5	7	9.5	14
Proximate children	37	2.08 (1.38)	0	.50	2	3	4
Number of frequent contacts	37	5.16 (2.47)	1	3	5	6.5	11
Illness work	37	16.6 (9.8)	2.78	10	15	20.9	43.3
Emotional work	37	30.0 (14.4)	1.67	18	29	39	65.0
**Self-management behaviours**							
SDSCA (scaled)	67	28.2 (9.19)	9	20	29	35	45
SF-12 (physical component scale)	67	50.1 (9.5)	32.4	42	50	58	68.4

Participants had an average of 7 to 8 members in their network, of which three were females. The average network consisted of: four different types of relationships with two children living nearby and frequent contact with five network members. Participants reported that network members provided them with a much higher degree of emotional work (support) than illness work. Participants reported high levels of self-care of diabetes (SDSCA). The mean of 50.1 for physical health status is relative to a standardised arbitrary mean of 50.0 for the wider sample.

### Causal beliefs

The most commonly ranked causal beliefs were: *genetics, diet and stress.*

### What participant characteristics and social network factors are associated with illness and fatalism beliefs?

None of the patient characteristics (Table 1) showed a relationship to any illness or fatalistic beliefs (P > 0.05 in all cases; results not shown) with the exception of number of chronic conditions, with participants with more than one condition perceiving more consequences of their diabetes (r = .341) (Table 3). A number of social network factors were associated with smaller concern about diabetes scores (i.e. less concern): a larger support network (*r* = −.470), greater frequency of contact (r = −.437), higher levels of illness (r = −.358) and emotional support (r = −.599), and a higher number of females in the network (r = −.366) (Table 3). Similarly, a larger support network (r = −.373), more frequent contact with network members (r = −.352), and greater emotional support (r = −.465), were related to less emotional distress over diabetes (Table 3). There were no significant relationships between the two fatalism variables and any of the social network variables, with the exception that fatalism 2 (little I can do to improve my health) and mix of network agents, non-significant in the initial analysis, became significant after removal of outliers (with greater fatalism associated with fewer agents: r = −.294; p < 0.05).

**Table 3:** **Illness beliefs, fatalistic beliefs, social networks**

BIPQ items	Consequences	Timeline	Personal control	Treatment beliefs	Identity	Concern	Understanding	Emotional distress	Fatalism 1	Fatalism 2
**Spearman’s Correlation Coefficient**										
Total number of conditions	.341**	.126	-.027	-.066	.122	.010	.030	.076	-.009	.143
No of supportive females	-.005	-.080	-.066	-.249	-.057	-.366*	.047	-.183	.002	-.259
Frequency of contact	-.193	-.146	.149	-.220	-.264	-.437**	.009	-.352**	-.212	-.309
Emotional work	-.171	.153	.241	.051	-.107	-.599**	**.306#**	-.465**	-.022	-.166
Illness work	.002	.238	-.183	-.209	-.010	-.358*	.146	-.195	-.278	-.300
Size of support network	-.276	-.088	.165	-.139	-.239	-.470**	.060	-.373*	-.258	-.298
Proximate children	.311	.232	.002	-.249	.099	-.055	-.006	.212	-.205	-.193
Types of relationships	-.150	-.215	-.035	.232	-.195	-.022	-.128	-.198	-.133	**-.294##**

*Significant at the 0.05 level (two-tailed).

**Significant at 0.01 level (two-tailed).

**# r = .368 (p < 0.05) ## r = −.358 (p < 0.05) became significant after removal of outlier(s).**

### Forward stepwise multiple regressions

Forward stepwise regressions were performed for the two BIPQ items which showed significant relationships with more than one social network factor in the previous correlational analysis: *concern beliefs, and emotional distress.* Concern beliefs: of the five social network variables (number of supportive females, frequency of contact, emotional work, illness work and size of the support network) entered into the regression, emotional work (r = .558) was the first variable to enter the regression. None of the remaining four variables reached the significance (P > 0.05). Emotional distress: of the three social network variables (frequency of contact, size of the support network and emotional work) entered into the regression, emotional work was again the only variable to enter the model as a significant predictor (r = .472) of emotional representations.

### How do illness and fatalistic beliefs relate to self-management behaviours and health outcomes in South Asians?

The following illness beliefs significantly correlated with better physical health (SF12): *consequences* (r = −.526), *personal control* (r = .348), *treatment control* (r = .398), *identity* (r = −.469), and *emotional representations* (r = −.336). Scores on the SDSCA were negatively correlated with *consequences* (r = −.309) and treatment control (r = .270) (Table 4).

**Table 4:** **Spearman’s correlation of illness and fatalism beliefs and self-management behaviours**

BIPQ	SF-12 (PNS)	SDSCA
Consequences	-.526**	-.309*
Timeline	-.124	.005
Personal control	.348**	.113
Treatment control	.398**	.270*
Identity	-.469**	-.137
Concern	-.278*	-.080
Understanding	.097	.257*
Emotional response	-.336**	-.103
Fatalism 1	-.114	.100
Fatalism 2	-.176	-.002

*Significant at the 0.05 level (two-tailed).

**Significant at 0.01 level (two-tailed).

### Nested qualitative study findings

Three themes from the nested qualitative study illuminate the quantitative findings. These are: fatalism; diabetes management - a family affair, and the use of alternative therapies. Data for each theme are presented as annotated extracts, along with participants’ gender, age, ethnicity and type 1 diabetes (T1D) or T2D, GP (P) or community participant (CP).

### Fatalism: cause and control

Analysis of the qualitative study revealed that many participants held fatalistic beliefs about diabetes:

*P: “Everything that happens is written in your fate” [P304, Pakistani, T2D]*

However, there were tensions between relating the cause of diabetes to fatalism and having knowledge about the scientific causes of diabetes. Similar to the quantitative study, participants referred to genetics, diet and stress as causes of their diabetes:

*P: “To me I’ve not eaten sweet foods or fatty foods because I have been fighting with my weight and still I’ve got it that means it were genetic because both my brothers’ got it. I think that’s what caused mine”. [P79, Indian, T2D]*

Believing that *God* controlled their diabetes was commonly expressed British South Asians with especially in the older first generation, migrant British South Asians. The British South Asian population in the UK can be sub-divided into first generation migrants and second generation British born [54]. The former group has received most attention in the literature yet the difference between the two generations is not always made explicit in research studies. The quantitative study did not distinguish between first and second generation British South Asians. But the qualitative study found that most of the participants were first generation migrants, and this information was revealed during the interview:

*“P: If something is going to happen, it’s going to happen, it doesn’t matter what the damn well you do….whether you control it, whether you don’t control it’s going to come” [P332, Indian, T2D]*

*“P: I am old now so there is no point worrying because nothing is going to happen by worrying. Whatever will happen God will decide, he sat here, the one who gave us life and he is the one that will decide. We have not come in this life with anything and nor are we going to be able to take anything with us, we will leave everything behind”. [CP2, Indian, T2D]*

Many of the first generation migrant British South Asians attributed the future of their diabetes, health and life to the time given by God:

*P: “It’s up to Allah, whenever I am going to die, I will die, and it’s when Allah decides my time is up. Even if I didn’t have all these illnesses I am going to die one day and even the youngsters die and the older ones stay behind. If death has to come it will come whether it’s a heart attack or an accident”. [P393, Pakistani, T1D]*

Whereas the British born South Asians attributed diabetes control to lifestyle factors such as exercise and diet rather than external factors such as God. These participants described taking more control of their diabetes by modifying their lifestyle and eating healthily and exercising to maintain good control of their diabetes:

*P: “We’ve changed the way we cook, and everything…The type of things we cook as well. So, less red meat and eat sensibly, loads of chicken, more vegetables and everything. I was…I’ve always been a bit chubby but not, you know, massive but it’s probably more diet than anything else…I exercise so… At the end of the day, you have to look after yourself” [P398, Pakistani, T1D]*

### Diabetes management – a family affair

Being aware of the high prevalence of diabetes in the community seemed to lead participants to normalise and downplay the seriousness of the condition. Having family members with diabetes seemed to help reduce the emotional distress of their own diagnosis:

*P: “Diabetes is in the family, my sister had it, my parents didn’t have it but other people in the extended family had it and I knew somewhere along the line it’s going to happen [CP34, Indian, T2D]*

Whilst findings from the quantitative study showed how greater emotional work from the network resulted in less concern and emotional distress related to diabetes, the qualitative study found that specific family members were cited as most important in tasks related to the management of medication and diet:

*P: “They (mother and sisters) make sure that they give me the right diet as well….They don’t give me much anything to do with sugars and things like that, so they make what I think is right” [P255, Pakistani, T2D]*

Although some participants held strong fatalistic beliefs about the cause and control of diabetes, they still reported attempts to change their lifestyles behaviours (e.g. diet) and share the responsibility of self-management with family members. For example, it was fairly typical that married men relied on their wife for managing their diet, food shopping and cooking for the family:

*P: “I think my wife knows about diets, and…she knows what affects me…and I think when she does the cooking, plans the cooking, the meal for the day, she always thinks about it. For example, one thing is cooking oil, we don’t use cooking oil, and we use olive oil”. [P134, Pakistani, T2D]*

*P: “She does the cooking - she is an expert cook…She plans everything, I just leave it to her, it’s her department and I don’t interfere [laugh]” [P96, Indian, T2D]*

Information on diet given to their wife was either in the form of written leaflets, or magazines taken from GP waiting rooms, or other information passed on from family and friends through conversation about diabetes. This was to ensure their wife knew what ingredients to buy to ensure they were eating the right foods for their diabetes:

*I: “How did your wife know about that? You know, like to change the oils and the diet and things?”*

*P: “It’s me, my influence, because I read a lot at the beginning….I told her what affects me…we talked about how to control it and…she’s got better, she likes cooking”. [P134, Pakistani, T2D]*

Some men also described how their wife or other females in their network, for instance their mother or sisters, provided support with their diabetes medication(s) and strongly believed that other people in the family should take responsibility for ensuring people with diabetes take their medication and eat the right foods:

*P: “My mother is very important because she’s always making sure wherever I travel I take my medicines with me and she rings up and asks ‘have you taken your medicine’…. my sisters are also very helpful…they also make sure that I do take them regularly….especially when I am travelling… there has to be someone who has to be on your head in order to make sure that you take the right medication…and right diet. [P255, Pakistani, T2D]*

In contrast to men, women with diabetes described feeling under pressure because of the responsibilities they had in the house in addition to managing their own diabetes:

*P: “Women especially they do have a lot of stress. We are running…we have two sides of the family and are pulled in all ways. You’ve got husband who wants this, his mother there and all parents. I think we have a lot of roles…you don’t have time…. I think also we don’t look after our diet a lot”. [CP21, Pakistani, T2D]*

For women, managing their diabetes, particularly their diet seemed to be secondary to the everyday work they had to do for their immediate and extended families. Some women reported on how their children helped to provide information and support related to diet. This was because the children were more competent in English and knew how to use the Internet:

*I: “Where or who do you find out about more about diabetes?”*

*Daughter: “It would be me or my brother”.*

*I: “Do you read any literature on diabetes?”*

*Daughter: “We translate it for her, so we read it to her…my mum doesn’t know much but my brother he likes to read…he has a laptop so he sits with my mum on the Internet and they look up things together”. [P304, Pakistani, T2D]*

A minority of participants who were fluent with English language described the use of email to exchange information on diabetes in order to learn how to maintain good control and reduce the risk of future complications:

*P: “We have conversations about it and my friend K actually sent me an email the other day about a diet regime he has tried….and yeah if somebody said that cut out the chapattis and eat more rice or vice versa then I’m quite happy to listen to that”. [CP34, Indian, T2D,]*

This highlights the tensions between fatalistic beliefs regarding diabetes control and future health, but at the same time trying to improve self-management and helping others to improve personal control:

*P: “I tell them because I want other people to realise that with diabetes you don’t have to suffer, you can control it and live a good healthy lifestyle and I like them to understand that if you eat healthy and look after yourself then you won’t suffer. So people get so many problems which slowly, slowly creep up”. [P79, Indian, T2D]*

### The use of alternative therapies

In the quantitative study, treatment control beliefs were significantly associated with better health status. However, the interviews revealed more and potential problematic information that did not have a strong evidence base. 'Alternative therapies’ (e.g. foods) were believed to improve diabetes control in conjunction with the GP prescribed allopathic medication. Karella (also known as ‘Momordica charanita’, or ‘bitter melon’) was the most common food supplement discussed. Participants believed that the bitterness from the vegetable Karella, would improve diabetes control by regulating sugar levels:

*P: “I have Karella and Karella bhaji they are very good. It brings the sugar level down yeah Karella bhaji and green chilli [laugh]. I have the aloe vera juice. My Pakistani friend says that Aloe Vera very small aloe vera…it makes the glucose levels go down because it is so bitter”. [CP5, Bangladeshi, T2D]*

Some participants also described taking a mix of herbs that were also believed to reduce blood sugar levels and improve diabetes control:

*P: “I’ve got my own remedy as well as the medical…so I use that….it’s a mix of herbs, which my wife usually does for me. It just reduces the blood sugar…it’s a mix of ginger, rosemary and wheat. In fact they said if you take it for 30 continuous…three months…continuous, all the time, it will get rid of your diabetes altogether. It’s a good remedy, it helps. …Honey and cinnamon as well, I’ve found it good for diabetes”. [P134, Pakistani, T2D]*

There was a stronger preference for food therapies made from so called natural ingredients, as these were believed to be less harmful to the body, with fewer side effects than the chemicals used to make allopathic medicines for diabetes:

*P:* “*Karella is a vegetable so if you eat there is no side effect it’s very good. I used to eat it a lot before and it worked”. [P326, Pakistani, T2D]*

*P: “I like to know a lot about herbal medicine…even before the diabetes I was always interested in that, because I think it’s a better alternative than the medicine we get from the doctor”. [P134, Pakistani, T2D]*

Participants reported that the frequent use of alternative therapies would help to one-day cure their diabetes, after reports about such therapies curing diabetes from other people in their community. This was reinforced by information informally exchanged and received from family and friends:

*P:"I think err….jamboora powder controls my diabetes better than the English medication. Using these medicines some people have permanently free of diabetes. [P296, Pakistani, T2D]*

*P: “Last time he (nephew) went to India, he told me about Jambu seeds, they are bitter and he brought me back the powder and I tried it. So if he finds out anything new he will tell me about it”. [P401, Indian, T2D]*

Few participants described the uncertainties about potential side effects of the certain food therapies, and the preference for these therapies was stronger for participants on oral medication than those on insulin injections:

*P: “I think that for people on insulin it probably doesn’t have much of an effect… whereas if its diet control you might be able to change it [CP38, Pakistani, T2D]*

## Discussion

### Summary of findings

This is the first study to explore associations between diabetes-related illness beliefs and sociocultural factors in British South Asians using mixed methods. Univariate and multivariate analysis of the quantitative study found that the key factors in predicting levels of diabetes concern and emotional distress (as measured by the BIPQ) were, larger networks, more frequent contactors, and higher levels of emotional work received, with the first two factors (size of the network and frequency of contact) appearing to operate mainly through their contribution to emotional work. The three qualitative themes: fatalism, diabetes management- a family affair, and use of alternative therapies; complemented, elaborated and extended some of the findings of the quantitative study. Whilst the results demonstrate the importance of sociocultural context in reinforcing the interpretation and meanings assigned to diabetes management, it also suggests that the CS-SRM and BIPQ lacks key elements to allow full understanding of illness beliefs in British South Asians with diabetes.

A common barrier in conducting mixed methods research is that of merging analysis of quantitative and qualitative studies to provide integrated analysis. To overcome this, we used the aims of the overall study to elaborate on the findings of both studies to show where the findings from both studies complement and contrast each other as intended when using this design [35].

In the quantitative study emotional work was a significant predictor of diabetes concern and emotional distress of diabetes; the more emotional support received within the network, the less concerned and less distressed British South Asians were about their diabetes. These findings lend some support to the previous social network literature on the availability of supportive ties in reducing emotional arousal in patients and guiding health behaviour [55]. Currently, within the CS-SRM, the dimension emotional distress neglects the role that others (social networks) may have in providing emotional support and mediating beliefs related to diabetes. Our quantitative study findings suggest that British South Asians may be dependent on their social networks, especially their family, for emotional support for their diabetes. Previous research into emotional distress suggests that cultural perceptions about the importance of family and social patterns of eating often contribute to anxiety and may hinder lifestyle modification for optimum diabetes control [56]. However, our qualitative study shows the importance of family members in assisting with diet control and lifestyle modification.

Causal and control beliefs were of special importance. The quantitative study indicated that participants ranked genetics, diet and stress as the three main causes of the onset of diabetes. The exploration of causal beliefs in the qualitative study revealed that some participants also attributed the onset of their diabetes to fatalism. This suggests that people who believe diabetes to be part of their ‘fate’ do so in the sense of it being genetic, as well as being ordained by God, and lends support to previous studies in the literature [7].

Participants with greater control beliefs had a better health status in the quantitative study. However, the findings of the qualitative study show that some participants believed that God and/or fate controlled their diabetes, with strong beliefs that family members should also take some responsibility for the management of their diabetes. As mentioned earlier, it is common for the findings in mixed methods to contradict one another. It has been argued that some South Asians do not believe the concept of control to be possible or desirable, as it is in Western societies [57]. Health and illness are often perceived as a balance between people and the supernatural world [58], and family members are often obliged to participate in performing care duties for the person living with an illness [7,57].

Participants with stronger treatment control beliefs were more likely to have better health status and engage in self-care behaviours. However, the question about treatment control in the BIPQ measure does not explicitly state the ‘type of treatment’. This is important as our qualitative study shows that it was common for British South Asians, especially migrants with T2D, to use 'food therapies' in conjunction with GP prescribed medication for diabetes. This finding lends support to the literature on the use of medicinal foods made from natural ingredients for the treatment of diabetes [59]. Participants with better health status, or higher levels of self-care behaviours reported fewer consequences related to diabetes. Although the quantitative study lends support to previous research on self-management behaviours and illness beliefs in South Asians [41], the qualitative study found that family members often took responsibility for tasks related to diet, medication and information provision. Thus, the SDSCA score in this study may not be a sensitive measure of self-care behaviours in this population and has to be treated with caution.

### Strengths and limitations

There are some limitations to this work - we obtained cross-sectional, self-report data which may be subject to social desirability and participants’ recall [60]. Although this is an efficient and cost-effective method of collecting data, the results cannot determine causal relationships. The measures used were not validated in the population under study, which may account for the non-normality of the data. For example, the fatalism item 1 confounds together two opposing explanatory mechanisms (chance and fate), hence some participants may have had difficulty understanding or deciding how to respond to the question and requires further validation.

Although the purposive sampling method increased the representativeness and size of the total sample, we had fewer participants complete the SNSI, in comparison to the questionnaire. Therefore the significant associations observed between the variables in the quantitative study, particularly from the multiple regression, should be treated with caution and need verification in further studies. It was not possible to establish validity of the measures, translate or use the existing translated measures (e.g. BIPQ) due to time and resource constraints. Thus, all questionnaires were presented to participants in English though they were clearly instructed to contact the research team for assistant with language support if necessary. Any missing information due to language comprehension was overcome with support from the interpreter, but some participants preferred to ask family members who were fluent in English to help with completing the questionnaire and interview. While the presence of the interpreter facilitated a good rapport it may have also influenced the data in that the interviewees’ responses may not have been captured accurately, and the interpreters may have found it easier to summarise the respondents’ answers to the questions asked, rather than interpret each answer verbatim [61].

Most of the participants (84%) had more than one chronic condition but this is true of older diabetic patients in general, though it does mean conditions other than diabetes may have influenced participants’ illness beliefs. The total sample comprised of participants with T1D and T2D but 90% of the sample had T2D and we believe that our findings principally relate to this group.

Despite the controversies over combining different research paradigms [33], the design used provided a wider conceptual scope to understanding of the statistical links between illness beliefs, social networks and gain a deeper understanding into the relationship between the psychosocial and cultural factors involved in diabetes management.

### Implications for policy, practice and theory

Given the support roles that appear inherent in British South Asian families, we recommend health service providers use strategies that challenge diabetes-related beliefs and include support and information for those involved in helping patients’ self-manage to reduce the burden on the National Health Service [62]. This would open up possibilities for both the person living with diabetes and for people in their social networks to have a common understanding of the tasks involved for effective diabetes control [63]. Developing education guidelines for health care practitioners [64] on how to account for diabetes-related beliefs in British South Asians using measures such as the BIPQ [17] may identify barriers to effective self-management. This could be a way to provide culturally sensitive care and inform behaviour change in primary care. Clinical practice may also benefit from further research into a direct comparison of diabetes-related illness beliefs and social networks between ethnic groups (e.g. South Asian and Caucasian).

Finally, our study shows that certain cognitive and emotional constructs are relevant to diabetes-related beliefs in the British South Asian population; therefore it may be worthwhile adding in constructs to the CS-SRM and BIPQ to make them more culturally sensitive to this target population and inform future interventions.

## Conclusion

Our study highlights the complex nature of sociocultural factors in shaping beliefs about diabetes management in British South Asians. Although the CS-SRM and subsequent measures have been influential in informing research and intervention in the general population, the findings of our study questions the ‘cultural sensitivity’ of the CS-SRM and BIPQ as they lack key elements to allow full understanding of key illness beliefs in British South Asians. The qualitative study illustrates the dominant influence of sociocultural context in reinforcing the interpretation and meanings assigned to diabetes self-management. Overall, the findings underline a need for health psychology to move beyond the notion of the ‘individual’ and account for the impact of sociocultural beliefs on self-management of diabetes. Future studies need to validate the appropriateness and feasibility of measuring contextual factors relevant to the experience of diabetes in South Asians.

## Additional file

Additional file 1: **Title of data.** Social network characteristics. Description: Table.

## Abbreviations

BIPQ: Brief illness perceptions questionnaire
CLAHRC: Collaboration of applied leadership in health and care people with long-term conditions
CS-SRM: Common sense self-regulatory model
GP: General practitioner
NHS: National health service
NIHR: National institute for health research
SDSCA: Summary of diabetes self care activities scale
SEM: Structural equation modelling
SF-12: Short-form 12
SNSI: Social network survey interview
SPSS: Statistical Package for the social sciences
T1D: Type 1 diabetes
T2D: Type 2 diabetes
