# Supplementary material for: Illness beliefs and the sociocultural context of diabetes self-management in British South Asians: a mixed methods study
Source: BMC Fam Pract. 2015 May 10;16:58. doi: 10.1186/s12875-015-0269-y (PMC4438635; doi:10.1186/s12875-015-0269-y)
Supplement: Additional file 1: — Title of data. Social network characteristics. Description: Table. [file 12875_2015_269_MOESM1_ESM.pdf]

Additional file

Appendix 1: Social network characteristics (*Vassilev et al, 2013*) [29]

Network characteristics	Description
Size of support network	A supportive network member was defined as being a member with a non-zero score on any of the work questions.
Frequency of contact	Members in contact at least weekly, including by phone, email or social media.
Number of supportive females	Number of supportive females in the network.
Proximate number of children	Children living in the same home or within a 5 minute walk/drive.
Mix of relationships (members in the network)	Number of different relationship types in the network (out of 10 types: *Immediate family, extended family, friend, neighbour, groups, work relationships, medical professionals, and other professionals, pet, other)*.
Emotional work	Assistance with providing comfort when worried or anxious about everyday matters, including health and well-being.
Illness work	Assistance with diet, medications, understanding symptoms, and making appointments.
